# A hematologic condition expressed as a lung disease

**DOI:** 10.12688/f1000research.1-28.v1

**Published:** 2012-10-12

**Authors:** Mónica Egozcue-Dionisi, José Nieves-Nieves, Ricardo Fernández-Gonzalez, Rosángela Fernández-Medero, Raúl Reyes-Sosa, José Lozada-Costas, Ramiro Pérez-Duardo, Román Vélez-Rosario

**Affiliations:** 1 Pulmonary Medicine Department, San Juan City Hospital, San Juan, Puerto Rico; 2 Hematology & Oncology Department, San Juan City Hospital, San Juan, Puerto Rico; 3 Pathology Department, University of Puerto Rico School of Medicine, San Juan, Puerto Rico

## Abstract

Pleural involvement secondary to Multiple Myeloma is considered a very rare complication. According to the literature only 1% of these patients develop a myelomatous pleural effusion. We present a case of a 39 year old man with multiple myeloma diagnosed six years prior to our evaluation, which developed progressive dyspnea, dry cough and right pleuritic chest pain two weeks prior to admission. On physical examination the patient had decreased breath sounds over the right posterior hemithorax accompanied by dullness to percussion. The chest radiogram was consistent with a right sided pleural effusion. Pleural fluid analysis revealed the presence of abundant abnormal plasma cells. The patient died four weeks after hospitalization. The presence of myelomatous pleural effusion is considered to be a poor prognostic finding, no matter at what disease stage it develops. So far no definite treatment has been shown to improve survival.

## Introduction

Multiple Myeloma (MM) is a malignant neoplasm characterized by abnormal proliferation of plasma cells. The disease is typically manifested by anemia, pathologic fractures, hypercalcemia and renal failure. Pleural involvement in MM is very rare and seldom has been described in the literature. To our knowledge, approximately eighty cases have been mentioned in the largest case series reported. Pleural effusions can be either myelomatous or non-myelomatous, the former being the less common presentation. Most cases of myelomatous pleural effusions are due to IgA MM. We present a case of a patient with a pleural effusion secondary to IgG MM.

## Case report

**Figure 1.**
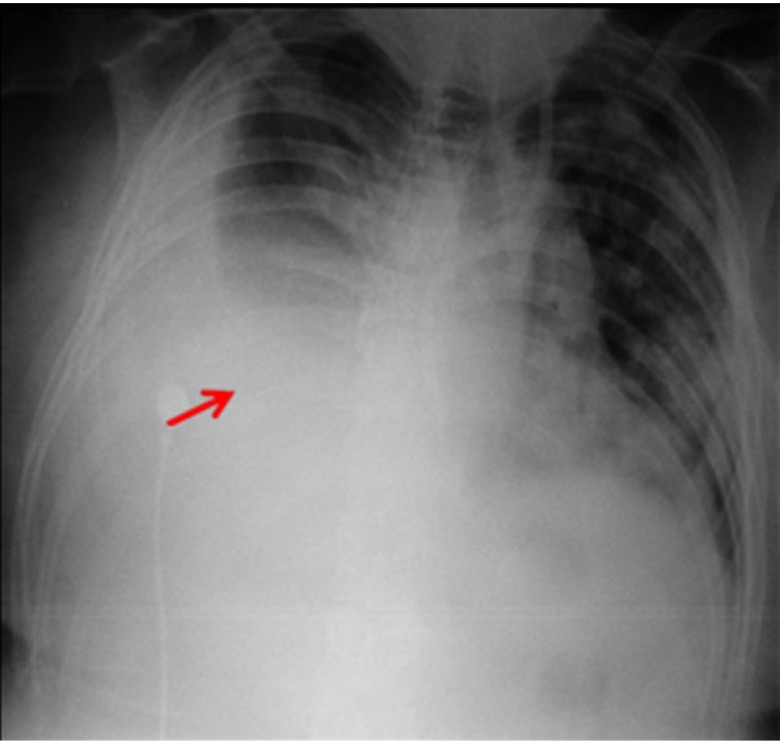
Antero-posterior chest x-ray on admission showing right-sided pleural effusion.

**Figure 2.**
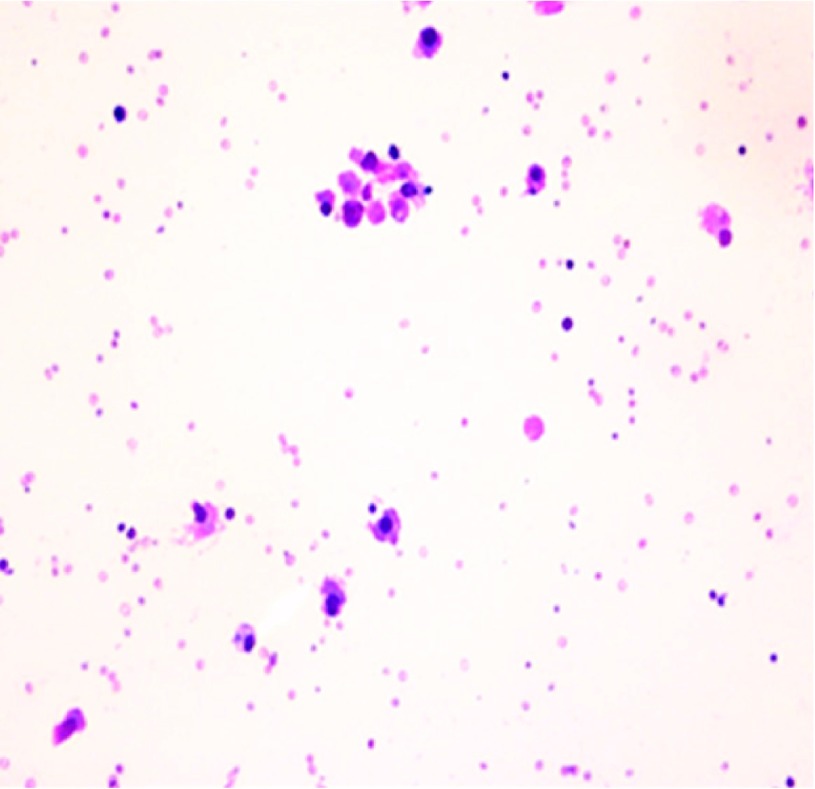
Photomicrograph of pleural fluid showing atypical plasma cells (Giemsa stain x 400).

A 39 year old man with hypertension, end-stage renal disease and chronic smoker, diagnosed with MM six years prior to our evaluation, came to our institution complaining of progressive dyspnea, fever, and dry cough of two weeks of evolution. He was treated with a course of oral antibiotics for five days with minor symptom improvement. On admission the patient was found with a temperature of 37.8°C, heart rate was 118/min., respiratory rate was 24 breaths/min., blood pressure was 120/74 mmHg, and oxygen saturation by pulse oximetry was 100% with a venturi-mask at 50% FIO _2_. Chest examination revealed multiple bilateral palpable plasmacytomas along the anterior and posterior hemithorax with decreased breath sounds below the right scapular area, and percussion dullness was heard on the right side. Antero-posterior chest radiogram showed a large right side pleural effusion with contralateral shifting of the mediastinal structures and patchy airspace opacities throughout the left lung ( Figure 1). Complete blood count revealed pancytopenia with a WBC of 2.8 × 109/l, Hb of 8.4 g/dl and platelet count of 19 × 10 ^9^. Blood chemistry showed a protein of 5.6 g/dl and lactate dehydrogenase of 721 IU/l. Calcium levels were within normal limits. Diagnostic and therapeutic thoracentesis was performed after platelet transfusion and a total of 900 ml of turbid, sero-sanguinous fluid was removed. Pleural fluid analysis was consistent with an exudate and the fluid cytology revealed the presence of abundant atypical plasma cells ( Figure 2). Bacteria, fungi and acid fast smear and cultures of the pleural fluid were reported negative. The patient’s clinical condition was aggravated by bacteremia, septic shock and respiratory failure requiring mechanical ventilation. The patient was complicated by sepsis and died four weeks after hospitalization.

## Discussion

Extra-medullary involvement in MM is considered to be a rare complication of the disease ^1^. Commonly involved sites are the nasal cavity, lung, pleura, thoracic wall, central nervous system, lymph nodes, spleen, skin and eyes. Involvement of serous cavities such as the pleural cavity, peritoneal cavity, cerebral-spinal space and pericardium is unusual, the pleural cavity being the most common site ^2^. Pleural effusions occur in 6% of the patients with multiple myeloma and can be myelomatous or non-myelomatous ^3– 5^. Non-myelomatous pleural effusions can occur secondary to sepsis, pulmonary embolism, chronic renal failure and secondary neoplasm ^6^. On the other hand, myelomatous pleural effusions have been described in only in 1% of the patients with MM and the diagnosis is based on the demonstration of monoclonal proteins in the pleural fluid by protein electrophoresis, finding monoclonal plasma cells in the fluid and/or histological examination of the pleura through biopsy ^7, 8^. Literature reveals that almost 40% of the cases of myelomatous pleural effusions are due to IgG type ^6^.

Multiple treatment regimens have been used including VAD regimen (vincristine, doxorubicin and dexamethasone), prednisolone, melphalan, etoposide, stem cell rescue and pleurodesis without a significant effect on mortality ^6, 9^. The use of bortezomib, a protease inhibitor, in refractory multiple myeloma has shown promising results. There is a single case of refractory MM and myelomatous pleural effusion treated successfully with intravenous and intrapleural bortezomib ^2, 10^.

## Conclusion

There has been limited information in the literature regarding pulmonary manifestations of this hematologic malignancy. Pleural effusions can be present as an initial manifestation of the disease or as the disease progresses. As in our case, pleural involvement is associated with poor prognosis and high mortality rate no matter at what disease stage it appears. So far, there are no proven treatment regimens that can halt disease progression. Physicians should be aware of such a fatal complication as it predicts a very poor outcome. For this reason, additional studies towards the development of new treatment strategies should be considered.

## Consent

Written informed consent for publication of clinical details and clinical images was obtained from the relative of the patient.
