# Supplementary material for: A hematologic condition expressed as a lung disease
Source: F1000Res. 2012 Oct 12;1:28. [Version 1] doi: 10.12688/f1000research.1-28.v1 (PMC3782346; doi:10.12688/f1000research.1-28.v1)
Supplement: Peer review history

# Peer review: Referee response for version 1

**Published:** 2012-11-12

**Authors:** Hermann Einsele

**Affiliations:** 1 Department of Internal Medicine II, University of Wurzburg, Wurzburg, Germany

The response quality following high‐dose chemotherapy and autologous stem cell transplantation for multiple myeloma (MM) is clearly correlated with progression‐free and probably also overall survival. Thus a major goal of all protocols for the treatment of younger patients with MM is to achieve a chest radiogram (CR). Recent data of the Spanish myeloma study group reveal long‐term disease free survival in a subgroup of patients with CR after autologous stem cell transplant (SCT).

Inclusion of novel agents (thalidomide, portezomib or lenalidomide) in the induction regimens prior to stem cell harvest and transplantation has allowed to achieve better remission quality prior to and after autologous SCT and to reduce treatment‐related mortality. Recently also protocols containing 2 novel agents have been used to improve induction regimens for patients with multiple myeloma.

In this study the PETHEMA/GEM group has studied and compared 3 induction regiments VTD (2 novel agents), TD and VBMCP/VBAD (an intensive regimen with bortezomib). VTD showed the best equality of remission (CR rate 35% vs 14% with TD and 21% with VBMCP/VBAD/B. Also the PFS after VTD plus autologous SCT was significantly longer when compared two both other induction protocols. Unfortunately 14% of the patients in the VTD arm developed grade 3 and 4 PNP. Thus, future studies should use either bortezomib or proteosome inhibitors with a lower neurotoxicity.

In addition, the VTD regimen in contrast to the GIMEMA study was not able to overcome the poor prognostic impact of high risk cytogenetics.Thus other treatment strategies e.g. allogeneic SCT, novel IMiDs or PI should be evaluated in these high risk MM patients (17p del, 7(4;14)).

I have read this submission. I believe that I have an appropriate level of expertise to confirm that it is of an acceptable scientific standard.

# Peer review: Referee response for version 1

**Published:** 2012-11-07

**Authors:** Sarah Waheed

**Affiliations:** 1 Myeloma Institute for Research and Therapy, University of Arkansas for Medical Sciences, Little Rock, AR, USA

The author has failed to mention if this patient had ever received any chemotherapy previously and if they did what agents were used. Also what was the initial presentation of myeloma in this patient?

The only imaging modality used was a Chest X-ray, it would have been helpful to know if a CT scan was done to evaluate if any pleural based mass was present along with the pleural effusion.

I have read this submission. I believe that I have an appropriate level of expertise to confirm that it is of an acceptable scientific standard, however I have significant reservations, as outlined above.

# Peer review: Referee response for version 1

**Published:** 2012-10-30

**Authors:** Artur Jurczyszyn

**Affiliations:** 1 Department of Hematology, Jagiellonian University Medical College, Krakow, Poland

I have read this submission. I believe that I have an appropriate level of expertise to confirm that it is of an acceptable scientific standard.
